# Regulation of the cell cycle and centrosome biology by deubiquitylases

**DOI:** 10.1042/BST20170087

**Published:** 2017-09-12

**Authors:** Sarah Darling, Andrew B. Fielding, Dorota Sabat-Pośpiech, Ian A. Prior, Judy M. Coulson

**Affiliations:** Cellular and Molecular Physiology, Institute of Translational Medicine, University of Liverpool, Liverpool L69 3BX, U.K.

**Keywords:** cancer, cell cycle, centrosomes, post-translational modification, ubiquitins

## Abstract

Post-translational modification of proteins by ubiquitylation is increasingly recognised as a highly complex code that contributes to the regulation of diverse cellular processes. In humans, a family of almost 100 deubiquitylase enzymes (DUBs) are assigned to six subfamilies and many of these DUBs can remove ubiquitin from proteins to reverse signals. Roles for individual DUBs have been delineated within specific cellular processes, including many that are dysregulated in diseases, particularly cancer. As potentially druggable enzymes, disease-associated DUBs are of increasing interest as pharmaceutical targets. The biology, structure and regulation of DUBs have been extensively reviewed elsewhere, so here we focus specifically on roles of DUBs in regulating cell cycle processes in mammalian cells. Over a quarter of all DUBs, representing four different families, have been shown to play roles either in the unidirectional progression of the cell cycle through specific checkpoints, or in the DNA damage response and repair pathways. We catalogue these roles and discuss specific examples. Centrosomes are the major microtubule nucleating centres within a cell and play a key role in forming the bipolar mitotic spindle required to accurately divide genetic material between daughter cells during cell division. To enable this mitotic role, centrosomes undergo a complex replication cycle that is intimately linked to the cell division cycle. Here, we also catalogue and discuss DUBs that have been linked to centrosome replication or function, including centrosome clustering, a mitotic survival strategy unique to cancer cells with supernumerary centrosomes.

## Reversible ubiquitylation

The post-translational attachment of ubiquitin moieties to substrate proteins, termed ubiquitylation, involves the covalent conjugation of ubiquitin, most commonly to lysine (K) residues. In the simplest form, ubiquitylation is the addition of an ubiquitin monomer, termed monoubiquitylation. However, the ubiquitin signal can be highly complex and is linked to a plethora of cellular processes [1,2]. Polyubiquitin chains linked through K48 target proteins for proteasomal degradation. However, ubiquitin possesses seven lysine residues (K6, K11, K27, K29, K33, K48 and K63) enabling the formation of diverse polyubiquitin chains that may be homotypic or heterotypic in nature, and can have alternative functions, as comprehensively reviewed in ref. [3]. The world of ubiquitylation is multifaceted and each layer relies upon families of proteins to write, read or erase this ubiquitin code. The steps to write the ubiquitin code are highly conserved, relying on an E1 ubiquitin-activating enzyme, an E2 ubiquitin-conjugating enzyme and an E3 ubiquitin protein ligase with substrate specificity. As reviewed in ref. [1], the human genome encodes two ubiquitin E1 enzymes, ∼40 E2 enzymes and >600 E3 ligases, a clear depiction of the complexity involved in functional ubiquitylation.

Ubiquitylation is a reversible post-translational modification, with removal of the ubiquitin signal catalysed by deubiquitylase enzymes (DUBs). The human genome encodes ∼100 DUBs that we refer to here as the DUBome (the cellular complement of DUBs). These DUBs belong to six families: the ubiquitin-specific protease (USP), ubiquitin C-terminal hydrolase (UCH), ovarian tumour protease (OTU), Josephin (JOS), JAB1/MPN/MOV34 (JAMM) families [4] or the newly discovered motif-interacting with Ub (MIU)-containing novel DUB (MINDY) family [5]. As reviewed in ref. [4], while most DUB families are thiol proteases harbouring a catalytic triad, the JAMM metalloproteases require a zinc ion to facilitate ubiquitin chain removal. As editors of ubiquitin signalling, DUBs are regulators of varied essential cellular processes, notably many have been assigned roles in DNA damage repair and cell cycle progression. As these processes are often dysregulated in cancer, DUBs, as potentially druggable enzymes, have quickly become the focus of several pharmaceutical companies vying to develop new cancer therapies.

## The cell division cycle

**Figure 1.**
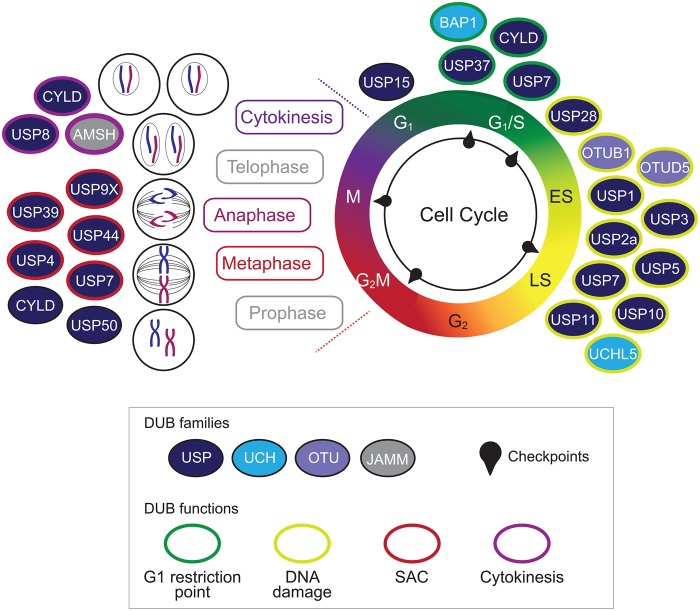
DUBs associated with the cell cycle. The cell cycle is schematically represented, highlighting key checkpoints and the individual stages of mitosis. DUBs with specific roles are indicated in the appropriate phases: solid colouring shows membership of the DUB families, and coloured edges illustrate the major cell cycle function. SAC: spindle assembly checkpoint.

The cell cycle co-ordinates cellular events to duplicate the genetic material and divide the cellular contents to create two identical daughter cells. The cycle comprises four stages. After division, cells undergo an initial growth phase (G_1_), followed by the replication of the genome (S-phase). A second growth phase (G_2_) prepares the cell for division and assembles cytoskeletal structures, before the genetic material divides between the daughter cells during mitosis (M). The unidirectional progression through these cell cycle phases is dependent on the periodic activation and inactivation of substrate proteins by kinases [including cyclin-dependent kinases (CDKs) and polo-like kinases (PLKs)] and ubiquitin-mediated degradation of key effectors by E3 ligases [including the APC/C (anaphase-promoting complex/cyclosome) and SCF (skp/cullin/F-box) complexes]. Accordingly, cell cycle effectors are regulated through protein–protein interactions, phosphorylation-dependent activation and ubiquitylation-dependent degradation, all working in concert to achieve an exquisite level of control throughout the cycle [6]. Once initiated, the cell cycle can be viewed as a series of autonomic cellular events that cascade until the eventual division into two daughter cells. However, checkpoints are inherent in the system, to temporarily halt the cell cycle if conditions are unfavourable. Many DUBs have direct or indirect roles during the cell cycle [7–9]. We discuss here selected examples of those regulating cell cycle progression and checkpoint maintenance, as summarised in Figure 1.

## DUBs and the G_1_ restriction point

Upon entering G_1_, cells are not committed to a subsequent round of cell division, as entry into the cell cycle requires sufficient mitogenic signalling to overcome a restriction point in late G_1_. Rb (retinoblastoma protein), the mediator of this restriction point, inhibits the E2F transcription factor during G_1_ [10]. Upon CDK4/6 activation by the G_1_-cyclin CCND (cyclin D) and then by CCNE (cyclin E), Rb is increasingly phosphorylated. This results in progression through the restriction point as hyperphosphorylated Rb dissociates from E2F, causing the transcription of S-phase genes [importantly CCNE and CCNA (cyclin A)] [11]. In addition to phosphorylation, Rb is regulated by ubiquitylation, being a target of the E3 ligase MDM2 [E3 ubiquitin protein ligase Mdm2 (double minute 2 protein)] [12,13], which promotes its proteasomal degradation [14]. The DUB USP7 directly antagonises MDM2-mediated polyubiquitylation of Rb, stalling the cell cycle in G_1_ [15]. USP7 is not the only DUB that may govern the restriction point, the tumour suppressor BAP1 (BRCA1-associated protein 1) also indirectly regulates the activity of E2F, via the deubiquitylation of HCF-1 (host cell factor 1), an important transcriptional co-regulator at E2F promoter sites [16,17]. CYLD (ubiquitin carboxyl-terminal hydrolase cyclindromatosis), another well-established tumour suppressor, plays a protective role during G_1_ via the transcription factor BCL-3 (B-cell lymphoma 3 protein). CYLD deubiquitylates BCL-3 inhibiting its nuclear translocation and so decreases the transcription of BCL-3 target genes including CCND [18]. CYLD therefore indirectly decreases CCND levels preventing cells from passing through the restriction point. During G_1_, the APC/C polyubiquitylates the S-phase cyclin CCNA, targeting it for degradation in order to prevent the cell from entering S-phase. The DUB USP37 directly regulates S-phase entry through antagonising activity of the APC/C^CDH1^ in G_1_ by removing polyubiquitin chains to stabilise CCNA [19].

## DUBs and DNA damage checkpoints

Key to successful cell division is maintaining the integrity of the genome during DNA replication in S-phase, and this is monitored by many quality control mechanisms. If DNA becomes damaged, checkpoints stall the cell cycle and activate DNA damage repair (DDR) pathways. This response revolves around p53 (cellular tumour antigen p53), which is stabilised and activated by DNA damage checkpoint signalling following a range of genotoxic insults. Under normal conditions, p53 is continuously synthesised but maintained at a low level by MDM2 polyubiquitylation targeting p53 for proteasomal degradation [20]. Under genotoxic stress, these regulatory mechanisms are reversed, to allow p53 to stall the cell cycle to enable repair or trigger apoptosis. At sites of DNA damage, sensors [e.g. 53BP1 (p53-binding protein 1)] facilitate the activation of DNA damage kinases [notably ATM (Ataxia telangiectasia mutated) and CHK2 (checkpoint kinase 2)], resulting in p53 phosphorylation. This abolishes the interaction between p53 and MDM2, increasing p53 levels and inducing transcription of p53 target genes [21], as well as activating transcription-independent roles of p53 in many of the major DDR pathways [22].

Given the integral role of p53 in cell cycle fate, it is perhaps unsurprising that many DUBs have been highlighted as direct or indirect p53 regulators, including USP2a, USP5, USP7 (HAUSP), USP10, USP11, USP28, OTUB1 and OTUD5. USP7, a predominantly nuclear DUB, was the first to be associated with the p53-dependent DDR via directly antagonising MDM2 polyubiquitylation of p53 [23]. However, USP7 also directly deubiquitylates the auto-polyubiquitylated MDM2, stabilising the E3 ligase as well as its substrate [24]. Although this may seem counterintuitive, USP7 exhibits a preference for MDM2 over p53 in unstressed cells, ensuring that p53 levels are maintained at a low level. Upon DNA damage, USP7 is dephosphorylated by PPM1G (protein phosphatase 1G) reducing activity towards MDM2, leading to increased auto-polyubiquitylation and degradation of MDM2, and the subsequent accumulation of p53 [25].

Other DUBs, including USP10, USP11 and OTUD5, also directly interact with, deubiquitylate and stabilise p53. Interestingly USP10, a predominantly cytoplasmic DUB, is involved in homeostasis of cytoplasmic p53 in unstressed cells, but, following DNA damage, a fraction of USP10 can translocate into the nucleus where it contributes to p53 activation [26]. As described for USP7, other DUBs indirectly control p53 levels via MDM2. For example, USP2a negatively regulates p53 levels through the stabilisation of MDM2, while exhibiting no deubiquitylating activity towards p53 directly [27]. OTUB1, another cytoplasmic DUB, can directly interact with p53, but predominantly stabilises p53 indirectly in the cytoplasm, through a non-catalytic mechanism. OTUB1 does this by binding and suppressing polyubiquitylation through the MDM2-associated E2 enzyme UbcH5 [ubiquitin-conjugating enzyme E2D 1 (UBC4/5 homologue, yeast)] [28]. In contrast, USP28 was shown to interact with and stabilise both the damage sensor 53BP1 and the checkpoint kinase CHK2 that activate p53 under genotoxic conditions [29]. USP5 uses perhaps the most indirect mechanism to stabilise p53 without physically interacting with components of the p53–MDM2 axis. It primarily disassembles unanchored polyubiquitin chains, and loss of USP5 results in accumulation of these chains that compete with ubiquitylated p53, but not MDM2, for proteasome recognition and degradation, so that p53 is selectively stabilised [30].

In addition, many DUBs have also been associated with executing specific DDR pathways [8]. For example, USP1 can support repair through both the Fanconi anaemia and translesion repair pathways [31]. An RNAi-based study has linked USP3 with double-strand DNA break repair; USP3 directly interacts with and removes monoubiquitylation from histones H2A (histone 2A) and H2B (histone 2B), and possibly other DDR effectors, to co-ordinate DNA repair [32]. Some DUBs exhibit a more global effect on DDR pathways, for example one screen revealed that UCHL5 was recruited to sites of DNA damage in addition to being involved in double-strand break resection [33].

## DUBs with roles in mitotic progression and cytokinesis

Following replication of the genome, and assuming checkpoints are satisfied in G_2_, the cell enters mitosis, where the newly replicated sister chromatids must be divided into each daughter cell. To achieve this, the cell passes through a sequence of distinct mitotic phases: prophase, metaphase, anaphase, telophase and cytokinesis (Figure 1). Prior to mitosis, the mitotic kinase CDK1 is held in an inactivate state by WEE1 (WEE1 G2 checkpoint kinase) phosphorylation, until SCF^βTrCP^-mediated ubiquitylation and degradation of WEE1 triggers mitotic entry; USP50 can repress mitotic entry through stabilising WEE1 [34]. Subsequently, USP7 can indirectly regulate the levels of Aurora A, a kinase required for correct maturation of the bipolar mitotic spindle, by stabilising CHFR (Checkpoint with Forkhead and Ring Finger), an E3 ligase that targets Aurora A for degradation [35].

USP44 was one of the first DUBs to be linked to mitotic progression, with a role in metaphase–anaphase transition [36]. Anaphase entry is stimulated by the APC/C and results in the separation of sister chromatids. To ensure the correct chromosome complement is distributed to each daughter cell, the spindle assembly checkpoint (SAC) monitors attachment of each chromosome pair to opposite poles of the mitotic spindle. Anaphase is arrested until the SAC is satisfied, preventing premature and inaccurate division of genomic content. Three key proteins, MAD2 (mitotic arrest deficient 2-like protein 1), BUBR1 (mitotic checkpoint serine/threonine-protein kinase BUB1 beta) and BUB3 (mitotic checkpoint protein BUB3), comprise the mitotic checkpoint complex (MCC) [37]. The MCC sequesters the APC/C activator CDC20 (cell division cycle protein 20 homologue) at unattached chromosomes, thus inhibiting the APC/C until chromosomes are correctly attached [37]. Once the SAC is satisfied, CDC20 is ubiquitylated and subsequently dissociates from the MCC to activate the APC/C [38]. USP44 plays a protective role at the SAC, directly antagonising CDC20 ubiquitylation, and so promoting MCC inhibition of the APC/C [36]. Once the SAC is satisfied, USP44 dephosphorylation decreases its activity towards CDC20, initiating mitotic exits through APC/C activation [39].

USP44 is not the only DUB that contributes to regulation of the SAC, for example USP39 and USP9X are also essential for the correct alignment of chromosomes at the mitotic spindle and their accurate division during anaphase. The mitotic kinase Aurora B is a key regulator of the attachment of sister chromatids to microtubules in the mitotic spindle. It exists in a complex with Survivin, the ubiquitylation status of which mediates interaction of the complex with chromosomes [40]. Depletion of USP39 results in decreased transcription and consequently lower levels of Aurora B kinase in cycling cells [41], while USP9X-mediated deubiquitylation of Survivin is required for dissociation from the chromosomes once correctly aligned [42]. Another DUB, USP4, plays an indirect role in the SAC through regulating correct splicing of mRNA transcripts, including for the mitotic checkpoint kinase BUB1 [43].

The DUB CYLD plays roles during both metaphase and cytokinesis. CYLD directly interacts with the catalytic domain of HDAC6 (histone deacetylase 6), inhibiting α-tubulin deacetylation and therefore indirectly increasing the stability of microtubules. This governance of microtubule stability by CYLD plays a role in spindle orientation during metaphase [44] and regulates the rate of cytokinesis [45]. Finally, USP8 and AMSH (associated molecule with the SH3 domain of STAM), two DUBs that are usually recruited to endosomes, have an important role in cytokinesis. The scission of the two daughter cells requires components of the ESCRT (endosomal sorting complexes required for transport) machinery including VAMP8 (vesicle-associated membrane protein 8), which co-localises with, and is deubiquitylated by, both USP8 and AMSH during cytokinesis [46].

## The centrosome cycle

Centrosomes are cytoplasmic organelles which act as the dominant microtubule-organising centres (MTOCs) in animal cells. During the cell cycle, centrosomes determine spatial arrangement of the microtubule arrays to influence cell shape, polarity, motility and organisation of the mitotic spindle [47,48]. The core components are two centrioles, small barrel-shaped organelles that are embedded in pericentriolar material (PCM). Each centriole consists of nine microtubule triplets arranged in a highly conserved rotational symmetry, imparted by SAS-6 (spindle assembly abnormal protein 6 homologue) during centriole assembly [49,50]. The PCM is a dense protein matrix composed of various proteins and exhibiting a high level of spatial organisation, and its major function is recruitment of γ-tubulin complexes which are essential for microtubule nucleation [51,52].

**Figure 2.**
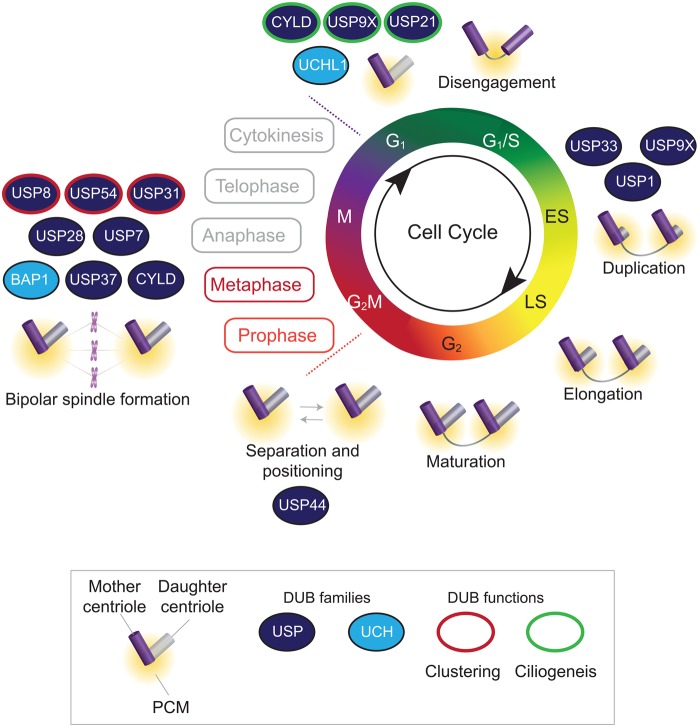
DUBs associated with the centrosome cycle. The cell cycle is schematically represented, highlighting the key stages of centrosome replication and function. DUBs with specific roles are indicated in the appropriate phases: solid colouring shows membership of the DUB families, and coloured edges illustrate the major function in the centrosome cycle.

Centrosome replication is strictly co-ordinated with cell cycle progression (Figure 2). Duplication of the single G_1_ centrosome begins at the G_1_/S transition and is completed during S-phase, so that two centrosomes are present in G_2_. These facilitate bipolar spindle formation at metaphase and are then segregated, one into each daughter cell, during cytokinesis [53,54]. Key to centrosome replication is centriole duplication, as the pre-existing mother centriole duplicates itself to form a daughter centriole. The kinase PLK4 and two SCF ubiquitin E3 ligases ensure that only a single replication event normally occurs. SCF^FBXW5^ ubiquitylates SAS-6 to target it for proteasomal degradation, preventing centriole over-duplication. SCF^FBXW5^ activity is limited by PLK4 to prevent premature SAS-6 degradation. Following G_1_/S transition, PLK4 homodimerises and *trans*-autophosphorylates, signalling recruitment of SCF^βTrCP^ which ubiquitylates and degrades PLK4. Decreased PLK4 levels restore SCF^FBXW5^ activity and block re-duplication [49,55–58]. Once duplicated, the daughter centriole elongates during S-phase and G_2_. This process is controlled by several genes including the multifunctional centriolar protein CP110 (centriolar coiled-coil protein of 110 kDa), which becomes ubiquitylated by SCF^cyclinF^ during G_2_ and mitosis. Centrosomes then undergo a maturation process, which requires recruitment of PCM. Finally, the centrosomes separate during G_2_, through KIF11 (kinesin-related motor protein Eg5) kinesin activity, which also facilitates bipolar spindle formation during mitosis [49,59,60].

Many human cells also display cilia in a cell cycle-dependent manner. During G_1_ (or G_0_ in terminally differentiated cells) centrosomes migrate to the cell cortex, where the mother centriole matures into a basal body which acts as a template for cilia elongation. During S-phase, both mother and daughter centrioles undergo duplication as normal. Then, prior to mitosis, cilia disassemble and the centrioles migrate back to the cell interior, ready to act as spindle poles during mitosis [61].

Various cell division errors, such as centrosome over-duplication, cytokinesis failure or cell fusion, can cause centrosome amplification, which is observed in many human cancers. The notion that, in addition to acting as MTOCs, centrosomes may function as signalling hubs [62] suggests one way in which amplification of centrosomes may benefit cancer cells. However, supernumerary centrosomes may cause multipolar spindle formation, impaired cell division, aneuploidy and genomic instability [63,64]. If uncorrected, multipolar spindles can lead to multipolar cell division and massive aneuploidy, which is usually lethal for the cell. Some cancer cells use mechanisms such as centrosome inactivation or centrosome loss to avoid multipolar divisions [65]. However, centrosome clustering is probably the most common response in cancer cells; this enables aggregation of additional centrosomes into two groups to form a pseudo-bipolar spindle and allow the cell to undergo bipolar cell division [66,67]. Ubiquitylation is increasingly recognised as a key regulator of centrosome biology [68], and our current knowledge of the role for DUBs in specific aspects of the centrosome cycle is summarised in Figure 2.

## DUBs regulating centrosome duplication and elongation during S/G_2_

During S-phase centrosomes must be duplicated exactly once. Many of the key proteins involved in centrosome duplication are ubiquitylated and therefore also open to regulation by deubiquitylation; the balance between these processes is imperative for precise duplication. For example, CP110 levels are normally tightly controlled during G_2_ and mitosis through ubiquitylation by SCF^cyclinF^, leading to CP110 degradation [69]. Countering this, USP33 localises to centrioles during S-phase and G_2_/M where it can deubiquitylate and stabilise CP110. Overexpression of either CP110 [70] or USP33 [71] leads to centrosome amplification. Similarly, appropriate expression of CEP131 (centrosomal protein of 131 kDa), a centriolar satellite protein, is required for accurate centrosome duplication [72]. Affinity purification and mass spectrometry identified USP9X as a CEP131 interactor [73]; USP9X localises to centrosomes in a cell cycle-dependent manner, most strikingly during S-phase and G_2_. USP9X gain-of-function leads to CEP131 deubiquitylation, stabilisation and centrosome amplification [73]. In addition, overexpression of a third DUB, USP1, is also linked with centrosome amplification. Although the mechanism remains unclear, USP1 may act, in part, through increasing expression of ID1 (inhibitor of DNA binding 1) [74], a fraction of which localises to the centrosome, as ID1 overexpression can induce centrosome amplification [75].

## DUBs affecting centrosome maturation, separation and mitotic spindle organisation during G_2_ and mitosis

BRCA1 (breast cancer type 1 susceptibility protein)/BARD1 (BRCA1-associated RING domain protein 1)-dependent ubiquitylation of γ-tubulin plays a key role in the regulation of centrosome duplication and microtubule nucleation, with BRCA1 loss resulting in centrosome amplification [76,77]. An siRNA screen for DUBs that affect levels of ubiquitylated γ-tubulin identified BAP1 and UCHL1 as candidates [78]. While UCHL1 interacts with γ-tubulin in G_1_, the BAP1 interaction is largely confined to mitosis, suggesting that these two DUBs regulate γ-tubulin in a cell cycle-dependent manner [78]. BAP1 removes ubiquitin from γ-tubulin, and mitotic defects in cells with low BAP1 levels are rescued by expression of BAP1 but not a catalytically inactive mutant. While the mechanism remains to be fully elucidated, it seems that deubiquitylation of γ-tubulin by BAP1 during mitosis allows proper spindle organisation and function [78]. CEP192 (centrosomal protein of 192 kDa) is a centrosomal protein with roles in maturation of centrosomes at the onset of mitosis and organisation of the mitotic microtubule landscape. Mass spectrometry identified the deubiquitylase CYLD as a CEP192 interactor, and CYLD co-depletion restores spindle assembly defects in CEP192-depleted cells [79].

In addition to its well described role in the spindle assembly checkpoint [36], USP44 also independently affects mitotic geometry by regulating centrosome separation and positioning [80]. USP44 interacts with CETN2 (Centrin2) and, although the targets of USP44 at the centrosome remain to be elucidated, catalytically inactive or CETN2-binding mutants of USP44 fail to rescue centrosome positioning defects. USP7 also plays a role in maintaining the correct number of centrosomes in a cell. It interacts with and stabilises centrosomal PLK1-phosphorylated 53BP1 at mitosis [81]. Depletion of 53BP1 results in lower levels of p53 and CENPF (centromere protein F), which is required for proper centrosome separation and spindle formation. Cells lacking 53BP1 accumulate supernumerary centrosomes, not through *de novo* amplification but rather due to failure of cytokinesis in cells with incorrect centrosome and spindle positioning and chromosomal missegregation. In contrast, USP37 depletion indirectly results in centrosome fragmentation, and hence multipolar spindle formation, through ubiquitylation and degradation of WAPL (Wings apart-like protein homologue), a regulator of sister chromatid resolution and spindle tension [82]. Notably, three recent papers have revealed a novel checkpoint, the mitotic surveillance pathway, that can detect centrosome loss or prolonged mitosis and results in cell cycle arrest [83–85]. The signalling pathway involves 53BP1 and the deubiquitylase USP28 acting in a complex to deubiquitylate and stabilise p53, which in turn controls cell fate.

## DUBs involved in centrosome clustering during cancer cell mitosis

Centrosome clustering is a mechanism that cancer cells containing supernumerary centrosomes commonly use to gather amplified centrosomes into two poles during mitosis, allowing for bipolar division and cancer cell proliferation [86]. Inhibition of centrosome clustering is an attractive, cancer-specific, therapeutic intervention. Two genome-wide screens have identified proteins required for centrosome clustering in *Drosophila* or human cells [67,87]. Analysis of the *Drosophila* dataset reveals prominence of proteins involved in ubiquitylation and the proteasomal pathway, including two DUBs, the *Drosophila* orthologues of human USP8 and USP31 [67]. The screen in human cells also identified USP54 [87], a DUB that is predicted to be catalytically inactive [88]. However, neither the ubiquitylation process nor these DUBs were investigated further in either study. In relation to its role in stabilising CP110 described above, USP33 may also indirectly affect centrosome clustering. Inhibition of CDK2 prevents CP110 phosphorylation that is required for centrosome clustering activity [89,90], and combining CDK2 inhibition with USP33 depletion has a co-operative effect on CP110, driving anaphase catastrophe via multipolar spindle formation [90]. In addition, the functional overlap of other DUBs with centrosome regulation makes it likely there are further DUBs involved in this process. For example, a functional SAC is required for effective centrosome clustering [67] and, as discussed above, several DUBs, including USP4, USP9X, USP39 and USP44, are required for SAC activity [36,41–43].

## DUBs involved in ciliogenesis during G_0_/G_1_

Many DUBs have been found to be required for the formation of primary cilia during G_0_/G_1_ phase of the cell cycle, a process termed ciliogenesis. Firstly, the DUB CYLD is recruited to centrosomes and the basal body of cilia via its interaction with CAP350 (centrosome-associated protein of 350 kDa), where it has to be present and catalytically active to promote docking of basal bodies at the plasma membrane and hence ciliogenesis [91]. A concurrent study also demonstrated that CYLD is required for docking of basal bodies at the plasma membrane and identified that this can, at least in part, be explained by its ability to deubiquitylate CEP70 (centrosomal protein of 70 kDa). Deubiquitylation of CEP70 allows it to interact with γ-tubulin at the centrosome to mediate ciliogenesis [92]. In addition, CYLD inactivates HDAC6, which modulates cilia length [92]. Secondly, via an independent mechanism to its roles in centrosome duplication, USP9X also regulates ciliogenesis [93]. During G_0_/G_1_, USP9X is recruited to the centrosome where it deubiquitylates and stabilises NPHP5 (Nephrocystin-5/IQ calmodulin-binding motif-containing protein 1), a positive regulator of ciliogenesis, so favouring cilia formation. However, at G_2_/M, USP9X becomes cytoplasmic, allowing degradation of NPHP5 and loss of cilia. Finally, a survey of DUB subcellular localisation found that USP21 localised to centrosomes and microtubules [94]. USP21 is required for effective microtubule regrowth from centrosomes, neurite outgrowth, generation of the primary cilium [94] and hedgehog signalling [95].

## Conclusions, future challenges and outlook

Here, we highlight specific roles for many different DUBs in controlling critical aspects of cell cycle progression, p53 homeostasis and DNA damage repair, as well as centrosome biology. To date, at least 30% of the DUBome has been associated with these processes, with predominant representation from the USP and UCH families. In addition to these roles, it is evident that many other DUBs regulate cellular processes during specific cell cycle phases. One example is the role of USP15 in regulating the transcriptional repressor RE1 silencing transcription factor (REST). Like many transcription factors, REST is rapidly degraded at G_2_/M prior to cell division; however, as it represses cellular differentiation genes, it must be reconstituted in G_1_. REST degradation is triggered by phosphorylation-dependent SCF^βTrCP^ ubiquitylation [96,97], and while this is reported to be antagonised by USP7 in neural progenitors [98], in cycling cells mitotic REST degradation appears to be unopposed. However, as cells exit mitosis, USP15 acts to deubiquitylate newly synthesised REST and rapidly rescue its expression levels [99]. Considering phase-specific roles such as this greatly expands the involvement of the DUBome in cell cycle biology.

This review aims to capture the current state of the field of DUB cell cycle research, but many outstanding questions remain. While certain DUBs have very distinct roles, others like CYLD, USP9X and USP7 play multiple roles at various phases of both the cell and centrosome cycles. Often we do not yet know how the function of a particular DUB is restricted to a cell cycle phase, or directed towards a specific target, to achieve precise temporal regulation of cell cycle effectors. Indeed, although transcriptomics suggest that USP1 is the only DUB that is periodically transcribed during the cell cycle [100], proteomics reveals periodic phosphorylation of several DUBs [101], but we at present lack a clear profile of regulated protein expression and activity for the DUBome during the cell cycle.

Although certain DUBs, OTUB1 being a notable example [28], play important roles through scaffolding interactions independent of their catalytic activity, most DUBs have catalytic functions. As highlighted in a recent review [102], unrestricted enzymatic activity of the DUBs would be hazardous for cells, and we are now beginning to appreciate the multi-layered mechanisms by which their activity can be controlled and directed. These include internal regulatory domains within some DUBs, interaction with allosteric regulators, incorporation into macromolecular complexes and post-translational modifications. Relevant examples for stabilisation of p53 in response to genotoxic stress include phosphorylation-dependent nuclear localisation of USP10 [26] and modulation of USP7 activity towards MDM2 [25]. Intriguingly, allosteric activation of USP7 by GMPS (guanine monophosphate synthase) that stabilises alignment of the catalytic site can also direct USP7 activity towards p53 under genotoxic stress [103,104]. These findings begin to rationalise the physiological roles of a DUB that is capable of stabilising both p53 and the E3 ligase MDM2, which targets p53 for degradation.

Another open question is why for certain processes, most notably in p53 regulation, there appears to be huge redundancy with multiple DUBs playing similar roles. One potential explanation is the ability of different DUBs to regulate p53 by different mechanisms and in different cellular compartments, as described above for USP7, USP10 and OTUB1. This may help ensure fine control of p53 activation in response to genotoxic stress. Critical roles for many DUBs have also been described in regulating the sharp and irreversible signalling decisions that are made at the G_1_ restriction point and the SAC. In both cases, a picture is emerging where DUBs contribute to a regulatory network, and each key component of the cascade is controlled by a specific DUB.

There is emerging interest in the role of the DUBome in centrosome biology, which less well studied than in the cell cycle. As a distinct organelle, it is easier to visualise how temporal roles for DUBs may be regulated, with some DUBs such as USP33 [71], USP9X [73] and BAP1 [78] already known to be recruited to the centrosome in a cell cycle-dependent manner. Where DUBs have been associated with the centrosome cycle, their mechanistic roles are often not yet well elucidated. For example, screens suggest that USP8 and USP31 may be linked to the centrosome clustering in *Drosophila* [67] and USP54 in human cells [87]. However, their centrosome-associated targets remain unknown, and no mechanism of action for these DUBs in regulating centrosome clustering has yet been suggested. It will be interesting to see whether DUBs predicted from screens and in model organisms do play significant roles in centrosome clustering in human cancer cells. Finally, in addition to acting as MTOCs, centrosomes have recently also been established as signalling hubs [62]. Many of the studies on DUBs at centrosomes we have discussed focus on their roles in duplicating and regulating the centrosome structure, and on their functions in nucleating microtubules. In future, it is likely that new roles for DUBs will be discovered in centrosome-based signalling pathways.

## Abbreviations

53BP1: p53-binding protein 1
AMSH: associated molecule with the SH3 domain of STAM
APC/C: anaphase-promoting complex/cyclosome
BAP1: BRCA1-associated protein 1
BCL-3: B-cell lymphoma 3 protein
BRCA1: breast cancer type 1 susceptibility protein
βTrCP: beta-transducin repeat-containing protein
CCNA: cyclin A
CCND: cyclin D
CCNE: cyclin E
CDC20: cell division cycle protein 20 homologue
CDH1: CDC20 homologue 1
CDKs: cyclin-dependent kinases
CEP131: centrosomal protein of 131 kDa
CEP192: centrosomal protein of 192 kDa
CEP70: centrosomal protein of 70 kDa
CETN2: Centrin2
CHK2: checkpoint kinase 2
CP110: centriolar coiled-coil protein of 110 kDa
CYLD: ubiquitin carboxyl-terminal hydrolase cyclindromatosis
DDR: DNA damage repair
DUBome: the cellular complement of DUBs
DUBs: deubiquitylases
ESCRT: endosomal sorting complexes required for transport
FBXW5: F-box/WD repeat-containing protein 5
GMPS: guanine monophosphate synthase
HDAC6: histone deacetylase 6
ID1: inhibitor of DNA binding 1
JAMM: JAB1/MPN/MOV34
JOS: Josephin
MCC: mitotic checkpoint complex
MDM2: E3 ubiquitin protein ligase Mdm2 (double minute 2 protein)
MTOCs: microtubule-organising centres
NPHP5: Nephrocystin-5/IQ calmodulin-binding motif-containing protein 1
OTU: ovarian tumour protease
p53: cellular tumour antigen p53
PCM: pericentriolar material
PLKs: polo-like kinases
Rb: retinoblastoma protein
REST: RE1 silencing transcription factor
SAC: spindle assembly checkpoint
SAS-6: spindle assembly abnormal protein 6 homologue
SCF: skp/cullin/F-box
UbcH5: ubiquitin-conjugating enzyme E2D 1 (UBC4/5 homologue, yeast)
UCH: ubiquitin C-terminal hydrolase
USP: ubiquitin-specific protease
WEE1: WEE1 G2 checkpoint kinase
